# Agricultural spider decline: long-term trends under constant management conditions

**DOI:** 10.1038/s41598-023-29003-2

**Published:** 2023-02-09

**Authors:** F. Samu, É. Szita, E. Botos, J. Simon, N. Gallé-Szpisjak, R. Gallé

**Affiliations:** 1 Plant Protection Institute, Centre of Agricultural Research, ELKH, Herman Ottó Street 15, 1022 Budapest, Hungary; 2 ‘Lendület’ Landscape and Conservation Ecology, Institute of Ecology and Botany, Centre for Ecological Research, 2163 Vácrátót, Hungary; 3 MTA-SZTE ‘Momentum’ Applied Ecology Research Group, Közép Fasor 52, 6726 Szeged, Hungary

**Keywords:** Climate-change ecology, Community ecology, Population dynamics, Zoology

## Abstract

There is widespread evidence for a worldwide trend of insect decline, but we have much fewer data about recent temporal trends in other arthropod groups, including spiders. Spiders can be hypothesised to similarly decline because of trophic dependence on insects and being equally sensitive to local and global environmental changes. Background trends in arthropod populations can be verified if we decouple large-scale environmental transitions, such as climate change, from local factors. To provide a case study on baseline spider community trends, we observed changes in the spider community of an unsprayed alfalfa field and its margin 23 years apart under largely unchanged local conditions. We aimed to determine whether there are changes in spider abundance, species richness and mean species characteristics. Spider abundance per unit effort decreased dramatically, by 45% in alfalfa and by 59% in the margin, but species richness and most characteristics remained unchanged. Community composition in both habitats shifted and became more similar by the current study period. The population decline was especially marked in certain farmland species. We propose that in the absence of local causative factors, spider abundance decline in our study indicates a reduction of spider populations at landscape and regional scales.

## Introduction

There is a growing concern about the scale of human induced worldwide species extinction, which is a signature of biotic change in the Anthropocene^1^. We have been relatively long warned about these trends, which affected invertebrates at least as much as vertebrate species^2,3^. This trend seems to be the most severe in relatively dry temperate regions^4^. Hallmann and co-workers’ study^5^ reported a striking 75% decline in flying insect biomass over a 27-year-long monitoring of nature protection areas in Germany. This study was accompanied by many other reports of insect biomass and diversity decline, e.g.^6–8^. A number of reviews analysed the worldwide trend and extent of insect decline, agreeing about the robustness of the process^9,10^ and pointing out its many facetted, but almost entirely human-related drivers^11–13^. Given the widespread evidence for insect decline, it would be important to know whether similar trends could be observed in another prominent arthropod taxon, spiders.

If we consider spiders’ ecological status, we expect similar trends in their abundance and diversity changes as in insects. Spiders are mostly generalist predators, and while they exhibit a diverse range of life styles, hunting modes and prey specialisation^14^, they mainly prey on insects^15^. Trophic dependence on insects leaves spiders vulnerable to the decline of their main prey group. Spiders are also affected by the same anthropogenic environmental changes and factors as insects^13,16^. Spiders are negatively influenced by climate warming^17,18^, habitat degradation^19^, habitat fragmentation^20^, biological invasion^21^ and management intensity in agricultural fields^22^ including pesticide use^23,24^.

While there is every reason to hypothesise that spiders are similarly affected as insects and exhibit a declining trend in their abundance and diversity, we have surprisingly few studies that address this question. Spiders were included but not detailed in an extensive German study, indicating a serious decline in biomass, abundance and number of arthropod species over a 10-year period^8^. A similar UK study notes that the decline in invertebrates other than insects was mostly driven by spiders^25^. Rix et al.^26^ assessed the long-term changes of trapdoor spiders in the family Idiopidae in agricultural areas of southern Australia. Their qualitative methods were mostly based on existence or lack of museum data, noting a “relative paucity of specimens and genera” in collections since 2014. Only a few quantitative long-term studies exist that explicitly address spiders. A study by Nyffler and Bonte^27^ evaluated population changes in the garden spider *Araneus diadematus* in Switzerland. The study relied on a single year but spatially replicated survey, where past and present locations were approximately the same. Compared to studies in the 1970’s the authors reported that present day population density of *A. diadematus* was only 0.7% of the historical values. An arctic study by Bowden et al.^28^ revealed that many of the dominant spider species’ abundances declined over an 18-year period, but no species increased in abundance in Northeast Greenland.

To gain a more robust, overall picture of spider population and diversity changes, long-term and geographically widespread studies would be necessary that include several climatic regions, habitats and different levels of human impact. This can be done only through a series of case studies. One possible strategy to gain knowledge about basic temporal trends, is to keep as many local parameters (e.g. habitat identity, management, methodology) constant as possible between surveys at different points in time. We aimed to provide such a case study by utilising and revisiting our own field survey from 23 years ago. We give an account of changes in the spider community over two decades, that is based on collecting in an identical habitat, with the same methods (even personnel). We compared data gained over more than one season and quantitatively considered not only one focal species but the entire spider community.

## Materials and methods

### Study area

We conducted sampling on the same arable field and its margin over two two-year periods, 23 years apart, in the seasons of 1996–1997 (past period) and 2019–2020 (current period). The 20 ha field was located on the area of the Experimental Station of the Plant Protection Institute, Centre for Agricultural Research, Nagykovácsi (N 47°32′54ʺ, E 18°55′59ʺ). In both the past and the current periods, the field had 2–3 years old alfalfa crop. A grassy perennial margin of variable width (c. 3–10 m) surrounded our study field. We conducted sampling in the alfalfa and the margin habitats. Sampling in alfalfa was performed close to the SE edge of the field, 10–50 m from the edge. The sampled margins were on the SE and SW sides. Regarding the field’s landscape context, in a 500 m zone around the field perimeter, on the NE and SE sides of the field there was a sparsely built up suburban area. On the NW and SW sides, there was a bushy and forested area mixed with abandoned hay meadows. The general landscape setting changed little between the study periods. However, notably close to the sampled margins, an alley of trees (*Acer platanoides*) has grown higher and crowns partly extended over the margin at the SE side, while on the SW side, in an unmanaged grassland close to the margin, bushes grew higher and became more dominant. Neither the field nor any surrounding area received intensive agricultural treatment in either period; specifically, there was no insecticide use on the field or in the margin.

### Sampling methods

In both periods, pitfall trapping and hand-held motorised suction sampling were used in parallel in both habitats. For pitfalls, we used 300 ml plastic cups of 75 mm upper diameter, filled with ethylene glycol–water solution (40%–60% by volume) as preservative. Suction sampling was performed with a modified leaf blower (Husqvarna 125bvx, petrol engine, 28 cm^3^ displacement) with 0.01 m^2^ suction nozzle^29^. We applied suctions in 10 consecutive separate press downs spread over a 10 m long transect, covering a total sample area of 0.1 m^2^. Each such suction transect was taken in the vicinity of a corresponding pitfall. Suction sampling and trap emptying was conducted at fortnightly intervals. In all four study years, we considered data from equivalent periods in the study years (20.05.1996–24.09.1996; 20.05.1997–16.09.1997; 21.05.2019–28.08.1019; 03.06.2020–09.09.2020).

### Experimental design

Pitfall traps were placed in linear sets of 3, 4 or 5 traps, with trap distances of c. 10 m both in the field and in the margin. Each suction sample (transect of 10 press downs) was taken in the vicinity of a concrete pitfall; this pairing resulting in that in each sampling set an equal number of pitfall and suction samples were included. Sampling sets were re-established every year at variable locations but at the same general area within the field or the margins. Table 1 gives the number of sampling sets deployed in each habitat and study year together with the total number of pitfalls included in the sets. Sampling sets comprised the basic units in the analyses to characterise abundance, species richness, species composition and different mean species characteristics of the spider assemblage. For this, data was cumulated over a year for all pitfall and suction transect data belonging to a given sampling set. The total yearly number of trap emptying and suction transects in a sampling set gave the sampling effort. Sampling effort was relatively homogeneous for all sampling sets (Mean ± S.D. = 50.1 ± 8.16). Detailed sampling effort data and resulting total catches are given, broken down by year and by habitat, in Table 1.

**Table 1:** Sampling effort data by habitat, study period and year.

Habitat	Alfalfa				Grassy margin			
Study period	Past		Current		Past		Current	
Year	1996	1997	2019	2020	1996	1997	2019	2020
Number of sampling sets	5	6	4	4	1	2	4	4
Total number of pitfalls	18	27	20	20	5	9	20	20
Total sampling effort	261	303	229	179	55	81	231	164
Total catch	3721	4721	1934	1476	450	1289	1323	857

Data from a fortnightly sample in a pitfall or data of a suction sampling transect of a total area of 0.1 m^2^ are treated as equivalent units of sampling effort.

### Identification and species characteristics

Adult spiders were identified to species, juveniles to genus or family level using available keys^30,31^. Abundance data in the analyses were based on all spider catches, both juveniles and adults, whereas data on species richness, mean species characteristics and community composition were only based on adult data. We used six characteristics of the spider species: body size, moisture preference, naturalness, rarity, hunting mode (web builder vs. hunter) and the status of being associated with arable habitats (agrobiont^32^ or farmland species vs. non-agrobiont species). The mean body size of each spider was a continuous variable in mm, based on literature data^30^. We followed Buchar and Růžička^33^ to characterise the moisture preference of spiders. Species were assigned with a specific value from 1 (preference for dry habitats) to a maximum of 5 (preference for moist habitats). Naturalness, i.e. preference for natural versus disturbed habitats, was similarly extracted from the work of Buchar and Růžička^33^. We gave values 1–4 to species based on their reported preference for artificial (1) to climax (4) habitats. Rarity was calculated from the extensive database of F. Samu, which contains spider ecological data based on catches nationwide in Hungary from 1993 to date in a wide range of habitats; for a general description of the database, see^34^. The database contains at present 90,233 records of 188,932 adult spider specimens belonging to 686 species. From this database, a relative rarity was determined against the most common species *Pardosa agrestis*, on a logarithmic scale giving rarity values ranging from 0 (most common, *P. agrestis*) to 10 (rarest species). Web building characteristics was determined based on general spider literature^30^ and was a binary character (web builder or non-web builder). Determining the agrobiont character of spiders (binary: agrobiont, non-agrobiont) was based on the list of core agrobiont species for Hungary^32^.

### Data analysis

For body size, moisture preference, naturalness, rarity, web building and agrobiont status we calculated community weighted mean (CWM, the average of species characteristics values weighted by the relative abundances of each species), with the “FD” package in R^35^.

We tested the effects of period (past, current) and habitat type (alfalfa, grassy filed margin) on spider species richness, abundance by general linear mixed-effects models (GLMMs)and CWM of the investigated species characteristics values by mixed-effect models (LMM) with the packages “lme4” and “MASS”^36^. We employed period and habitat type as categorical predictor variables and year as a random factor to account for the temporal non-independence of samples within periods. To account for differences in the number of sampling units per sampling set, we included the log-transformed sampling effort as an offset variable in our models^37^. We used Poisson error term for species richness and abundance models. We assessed overdispersion by comparing the residual deviance to the residual degrees of freedom. Overdispersion was detected in case of abundance. Therefore, we recalculated the models with negative binomial error term. We used Cook’s D distance to measure the influence of an observation on the estimation of the coefficients. We treated observations with D > 0.5 as influential observations and excluded them from further analyses^37^.

We studied the multivariate response of spider communities to period and habitat type with Permutational Multivariate Analysis of Variance (PerManova) using Bray–Curtis distance matrices in the “vegan” R package^38^. The analysis was based on a Bray–Curtis dissimilarity matrix with 5000 permutations. To visualise the community response we have separately ran a PCA analysis with the Canoco 5.15 software^39^.

## Results

**Figure 1:**
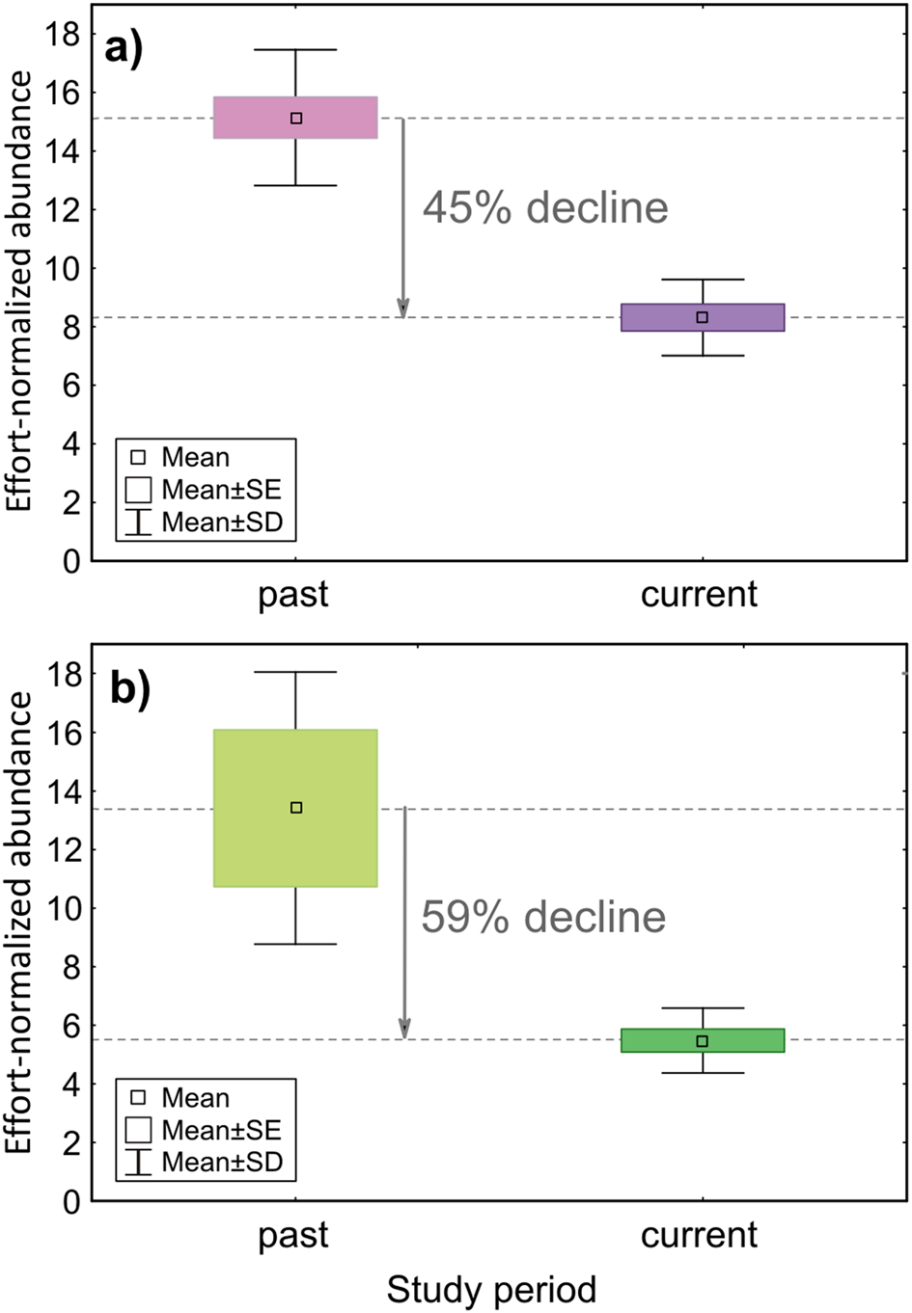
Change of abundance per unit sampling effort between past (base line) and current period in (**a**) alfalfa and (**b**) grassy field margin.

During the whole study we have collected 18,459 spider individuals, out of which 8007 were adults, belonging to 127 species. Species list and total catches by period and habitat are reported in Appendix Table 1. We found the strongest effect of period on the abundance of spiders. In the past period we caught significantly more spider individuals per sampling set than in the present period. This effect was relatively more pronounced in the margin habitat. Considering the mean number of individuals caught per unit sampling effort over the two seasons of a study period, in alfalfa the current effort-normalised abundance showed a 45.1% decline from the baseline in the past, whereas in the margin habitat this decline was as high as 59.1% (Fig. 1a,b).

**Figure 2:**
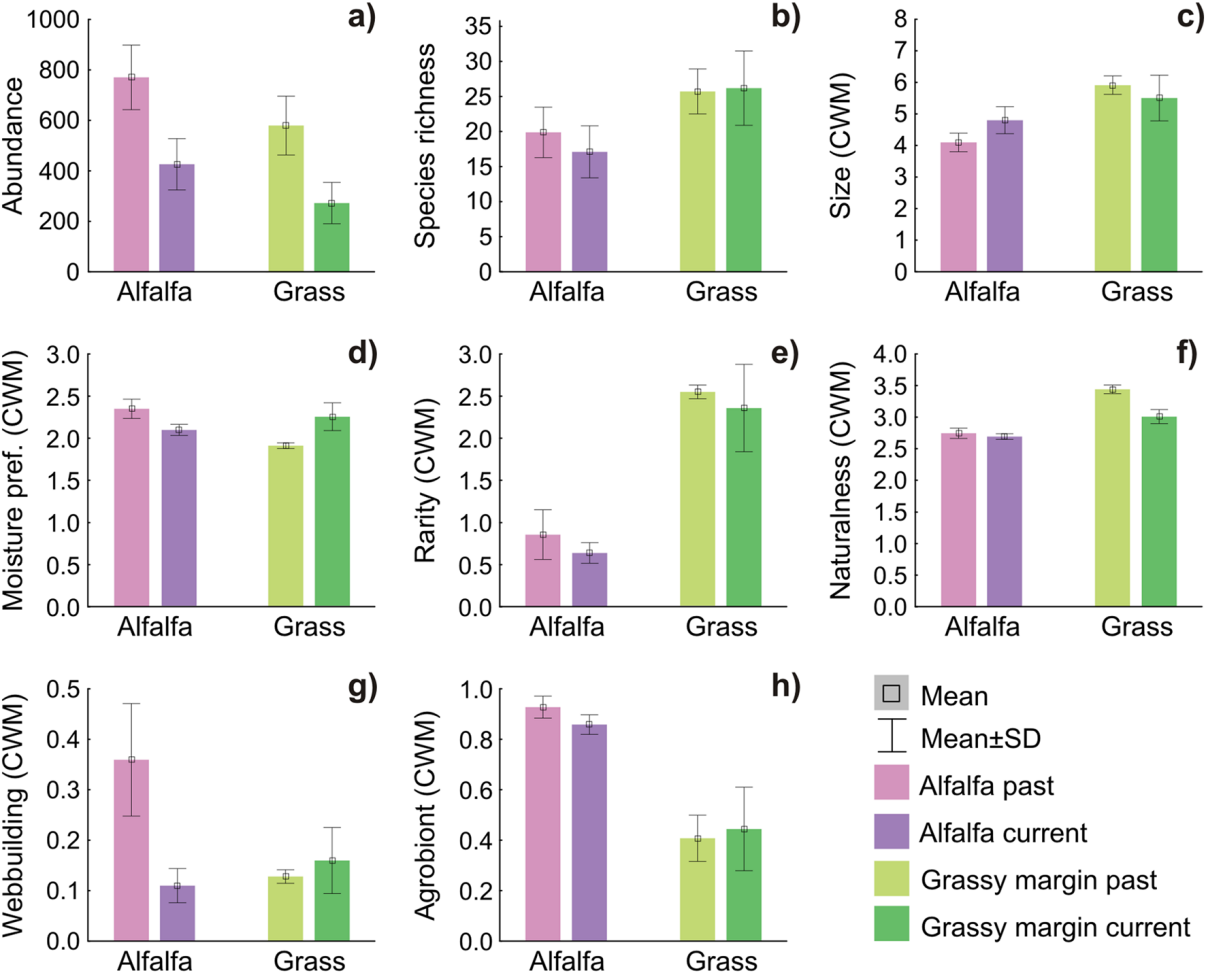
Habitat type and sampling period affect spiders (mean ± SD). Darker shades represent the current period; lighter shades indicate the past sampling period. (**a**) total abundance, (**b**) species richness, (**c**) CWM of spider size, (**d**) CWM of moisture preference, (**e**) CWM of rarity, (**f**) CWM of naturalness; (**g**) CWM of web building hunting strategy; (**h**) CWM of agrobiont status. Refer to Table 2 for further details.

However, we found no difference between the periods in the case of species richness (Table 1, Fig. 2a,b). There was also a significant habitat effect: we found higher spider abundance in alfalfa and higher species richness in the margin. We collected larger spiders in the current period than in the past in alfalfa fields. However, we found the opposite pattern in the grassy field margins; spiders were smaller in the current period than in the past (Table 1, Fig. 2c). The spider community had on average a higher moisture preference in the past in alfalfa, but in the margin the opposite case was found with more moisture preferring spiders in the present period (Fig. 2d, Table 1). Both rarity and naturalness were higher in the grassy field margin, and naturalness decreased over time in the grassy field margins, but not in the alfalfa fields (Table 1, Fig. 2e,f). The ratio of web builders was significantly higher in alfalfa and it also significantly decreased with time there, as opposing to an increasing trend in the grassy margin (Table 1, Fig. 2g). There were more agrobionts in alfalfa than in the margin habitat (Table 1, Fig. 2h), but no temporal trend could be shown for this species characteristics.

**Table 2:** Results of the LMM and GLMM models.

	Period (past/current)	Habitat (alfalfa/margin)	Period: habitat
Abundance^a^	− 0.597 (− 6.892)***	− 0.416 (− 4.476)***	0.294 (1.983)***
Species richness^b^	− 0.118 (1.056)	0.462 (4.194)***	− 0.507 (− 0.291)
Size^c^	0.679 (2.898)**	0.743 (3.008)**	1.171 (2.867)**
Moisture preference^c^	− 0.264 (− 1.613)	0.196 (2.535)*	− 0.540 (− 4.200)***
Rarity^c^	− 0.199 (− 0.734)	1.813 (10.000)***	0.069 (0.229)
Naturalness^c^	− 0.055 (− 0.257)	0.360 (4.888)***	0.442 (3.604)**
Webbuilders^a^	− 0.113 (0.823)	0.275 (4.21)***	− 0.244 (− 2.264)***
Agrobionts^a^	0.297 (1.809)	0.122 (2.260)*	− 0.138 (− 1.554)

Estimates (z/t values) are given.

We included sampling year as random effect and sampling effort as offset in all models.

Significance levels: *p < 0.05; **p < 0.01; ***p < 0.001.

^a^Model fitted with negative binomial error term (GLMM).

^b^Model fitted with Poisson error term (GLMM).

^c^Model fitted with Gaussian error term (LMM).

**Figure 3:**
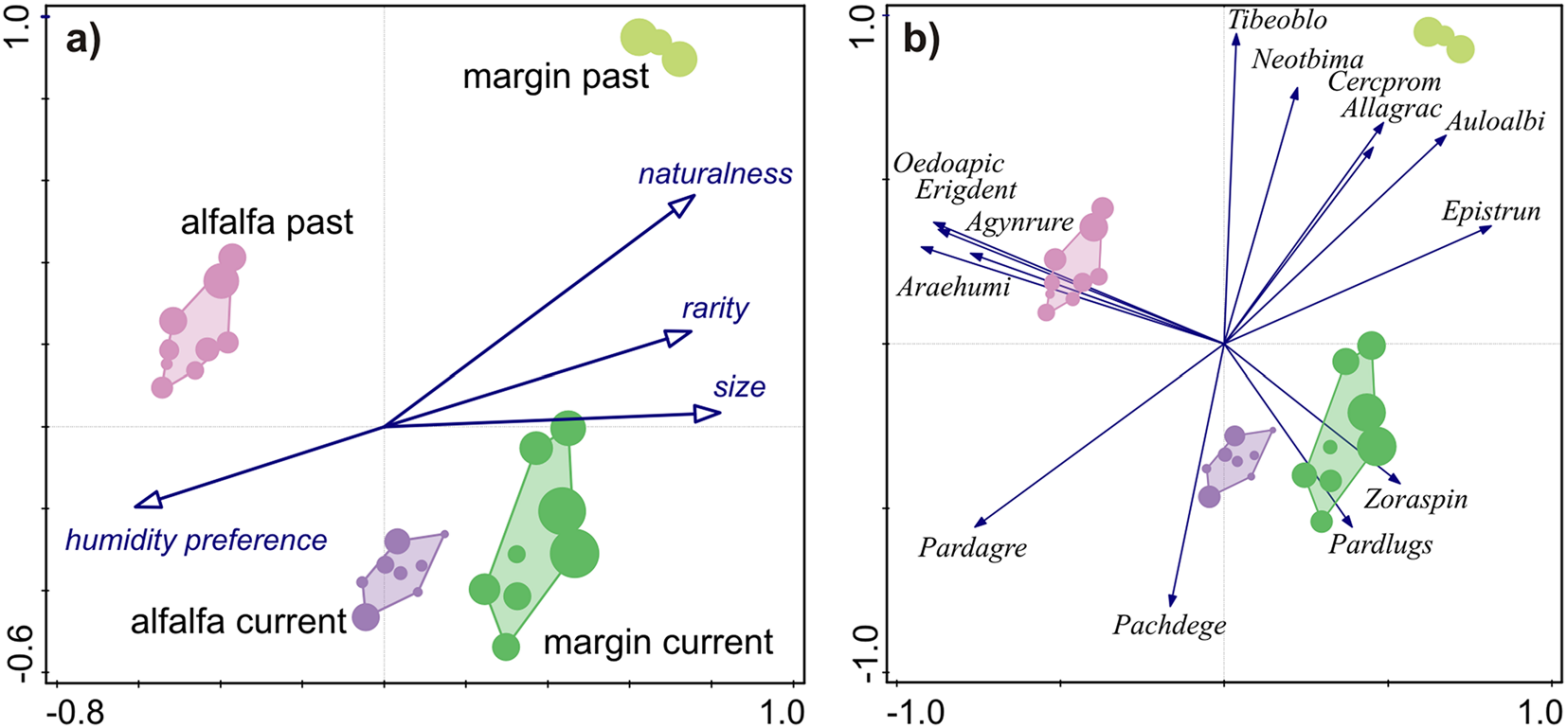
Unconstrained ordination (PCA) of sampling sets in the two studied habitats belonging to the current or past study period (identity of sampling set groups is colour coded and labelled on the figure. Dot size indicates species richness of the sampling sets. (**a**) Community weighted mean species characteristics as supplementary variables are depicted as a biplot; (**b**) the most influential species are depicted as a biplot on the PCA diagram. Species abbreviations are as follows: Agynrure—*Agyneta rurestris*; Allagrac—*Allagelena gracilens*; Auloalbi—*Aulonia albimana*; Cercpromi—*Cercidia prominens*; Epistrun—*Episinus truncatus*; Erigdent—*Erigone dentipalpis*; Neotbima—*Neottiura bimaculata*; Oedoapic—*Oedothorax apicatus*; Pachdege—*Pachygnatha degeeri*; Pardagre—*Pardosa agrestis*; Pardlugu—*Pardosa lugubris* s.str.; Tibeoblo—*Tibellus oblongus*; Zoraspin—*Zora spinimana*.

There was a significant effect of study period (R^2^ = 0.182, F = 12.245, p < 0.001), habitat type (R^2^ = 0.306, F = 20.546, p < 0.001) according to the PerManova model. Their significant interaction (R^2^ = 0.122, F = 8.198, p < 0.001) revealed that the effect of habitat was different in the two study periods on the community structure of spiders, or, equally, the temporal effect was different between habitats. These findings were visualised by ordinating sampling sets in the species space by PCA analysis (Fig. 3). The first two axes accounted for the cumulative variation of 32.6% and 56.9% respectively. Sampling sets were distinctly separated by study period and habitat, with community changes in both habitat types occurring in the same direction, indicating a temporal gradient. The habitat × study period interaction was also clearly visible on the ordination plot, as the spider communities of the two habitat types became more similar by the current study period. Depicting community weighted mean species characteristics as supplementary variables on a biplot (Fig. 3a), revealed that while supplementary variables accounted for as much as 45.1% adjusted explained variation, their vector was mostly perpendicular to the temporal gradient, therefore predominantly explained between habitat variation. A species–sampling set biplot (Fig. 3b) showed that individual species reactions could be associated with the temporal changes between the past and current study periods.

## Discussion

We observed a clear declining trend in the studied spider populations. In both the field and the margin habitats, over two decades, there was a substantial decrease in spider abundance but no such change in species richness. Community composition changed considerably in both habitats, but most notably spider communities in the margin and field habitats became more similar by the current observation period. The examined species characteristics, such as body size, humidity preference, rarity, hunting strategy and agrobiont status, changed in a complex way. Habitat type more than study period determined differences in mean species characteristics, with temporal changes occurring in some instances to different extent or even different directions in the two examined habitats. The most robust phenomenon—spider abundance decline—largely agrees with the changes observed in the few other available studies, either addressing a single spider species^27^ or the community level^8^.

Documenting temporal trends in spider abundance and diversity is important, because there is a paucity of data about most invertebrate groups, and our assessment of diversity and abundance changes is biased towards a few well-studied taxa^40^. Further, as opposed to presence-only occurrence records, multi-species trends allow a more realistic view of current trends^25,26^. The original presumption of the present study was that spider populations are likely to follow insect populations because of the strong trophic links^41^, and also because they are sensitive to and respond similarly to environmental changes as insects and other terrestrial invertebrates^13^. Our estimation of 45–59% decline in spider abundance over 23 years corresponds with or rather positioned at the lower end of similar figures for insects or invertebrates in general. A meta-analysis of invertebrate abundances indicated a 45% reduction across two-thirds of the taxa world-wide^42^, although for tropical regions 78–98% losses in arthropod biomass were also shown^43^. In German grasslands mean arthropod abundance decline amounted to 78%^8^, and a 76% decrease in flying insects over 27 years in German protected areas was indicated in another systematic survey^5^.

Contrary to the abundance decrease, in the surveyed alfalfa field and margin spider species richness did not decrease significantly. In terms of species characters, there were mixed responses. On one hand, we could not observe general shifts in characteristics that indicate the quality of the communities, such as rarity, naturalness, ratio of agrobiont species and the aforementioned species richness. Neither could we detect change in humidity preference, which is a characteristics that could indicate climate change induced effects. On the other hand, some other characters changed significantly, such as body size and the prevalence of web-building, but with strong habitat and interaction effect, meaning that we had different changes over time in the two examined habitats.

We argue that many of the changes in community weighted mean characters were due to specific species responses, which—as revealed by the ordination analysis—resulted in considerable shifts in species compositions over time. Species turnover may result in an overall unchanged species richness, but also in changes in mean character values. The apparent contradiction between abundance decline and a maintained species richness was also found in an arctic survey of spiders, where during a monitoring period of 18 years the number of species did not change, but in some species abundances declined and no species increased^28^.

In our case species responses were more varied. Some spider populations either increased or decreased. Species turnover was the most pronounced in the case of species found in low numbers. In the past survey period, in alfalfa many of the core agrobiont spiders^32^ could be observed with high abundances that decreased considerably in current alfalfa catches (e.g. *Agyneta rurestris* from 463 to 72 individuals; *Oedothorax apicatus* from 260 to 1; *Erigone dentipalpis* from 446 to 8). In current alfalfa catches other agrobiont species roughly maintained their dominance, like *Pardosa agrestis* (2324 past, 1560 current) or *Pachygnatha degeeri* (85 past, 149 current). These changes might explain why web-builder ratio decreased in alfalfa, since most of the agrobionts that declined were web-builder spiders from the Linyphiidae family, while other agrobionts that expressed only a moderate reduction or even increased were hunting species (adults of *Pachygnatha degeeri* were counted as hunters^44^). The ordination revealed that a number of species, which were associated with the margin habitat in the past, decreased or disappeared in current margin catches. *Aulonia albimana* (decreased from 290 individuals to 37) and *Tibellus oblongus* (decreased from 264 to 2) could be considered as specifically ecotone species frequent at the border of agricultural and semi-natural habitats^45^. At the same time, other spider species increased substantially in current margin catches, such as *Pardosa lugubris* (from 0 to 290 individuals) and *Zora spinimana* (from 1 to 30), both species having an association with leaf litter^46,47^, likely indicating the fact that the habitat on the non–field side of the margin became more wooded; bushes and trees had grown over the observation period. The overall trend of decreasing dominance of margin specialists in the margins and decrease of farmland specialists in alfalfa resulted in the significant homogenization process revealed by the multivariate analyses. Despite such shifts, species turnovers in both directions seemed to balance each-other, resulting in an apparent overall stability in species richness. However, the serious decline in spider abundance warns that decreasing population sizes of individual species precede local extinctions and an overall loss of biodiversity^13,43^.

We have documented a considerable decline in the abundance of all spiders and even relative to that a decline in the ratio of certain farmland species. Such processes, which are likely to occur also at larger scales, may have negative implication, considering the biocontrol potential of spiders^14,48^, but also considering the general loss in biodiversity and the stability of farmland ecosystems. We also might ask why do we experience this decline if the local habitat remained relatively unchanged and management was largely the same over the study span? The likely answer is that wider scale processes, such as climate change and land-use intensification could have significantly contributed to the observed phenomena. Under continental climate and in landscapes dominated by agricultural use, it is hard to disentangle these two effects. A manipulative field-scale experiment that intended to study the simultaneous and separate effects of land-use intensification and climate change, found parallel overall effects of the two factors, manifesting mainly in abundance reduction of arthropods in the manipulated grassland plots^49^. Climate change alone have been documented to dramatically influence arthropod communities, including spiders, in natural biotopes^18,50^ and in especially vulnerable climatic regions, such as in the tropics^43^ and under arctic climate^28,51^.

Land-use intensification, such as management intensification, less-diverse crop rotation and the loss of semi-natural habitats are alone significant drivers of arthropod decline^13^. Complex landscapes comprise a mosaic of different habitats, supporting high overall biodiversity^52^, but landscape simplification results in a loss of farmland biodiversity^53^. Such processes are also catalysed by socio-economic drivers, such area based subsidies resulting from some EU CAP programmes^54^. Periodically disturbed agricultural habitats^55^ are distinctly exposed—through meta-population dynamics—to the wider scale condition of its constituent populations^56,57^. Therefore, arthropod decline at the local (field) scale is largely influenced by changes at the landscape scale. Our study sites did not change regarding local management intensity, but at a regional level the above mentioned landscape level processes were significant, with field sizes increasing, margins, natural habitat patches decreasing. We think that changes in the spider populations, documented in the present study, are indicators of wider scale changes either or both regarding climate and regional land-use change. Such case studies widen the taxonomic coverage of documenting arthropod population trends and will allow future meta-analyses to identify the robustness of such trends, including disentangling their causative factors, and possibly giving hope for their mitigation.

## Supplementary Information

Supplementary Table 1.

## Data Availability

Basic data are available in Appendix Table 1. The datasets generated during and/or analysed during the current study are available from the corresponding author on reasonable request.
