# Supplementary material for: Agricultural spider decline: long-term trends under constant management conditions
Source: Sci Rep. 2023 Feb 9;13:2305. doi: 10.1038/s41598-023-29003-2 (PMC9911793; doi:10.1038/s41598-023-29003-2)
Supplement: Supplementary file 1 — Supplementary Table 1. [file 41598_2023_29003_MOESM1_ESM.docx]

## Supplementary information

“Samu, F., Szita, É., Botos, E., Simon, J., Gallé-Szpisjak, N., Gallé, R.: Agricultural spider decline: long-term trends under constant management conditions”

**Appendix Table 1.** Total catches of adult spiders in the alfalfa and margin habitats over the the past and current study periods.

| \|  \| **alfalfa** \| \| **grassy margin** \| \| \| --- \| --- \| --- \| --- \| --- \| \| **Species** \| **current** \| **past** \| **current** \| **past** \| \| Agelena labyrinthica (Clerck, 1757) \|  \|  \| 1 \|  \| \| Agyneta rurestris (C. L. Koch, 1836) \| 72 \| 463 \| 43 \| 27 \| \| Agyneta similis (Kulczynski, 1926) \| 1 \|  \|  \|  \| \| Agyneta simplicitarsis (Simon, 1884) \| 69 \| 8 \| 21 \| 2 \| \| Allagelena gracilens (C. L. Koch, 1841) \|  \|  \|  \| 2 \| \| Alopecosa cuneata (Clerck, 1757) \|  \| 1 \|  \|  \| \| Alopecosa pulverulenta (Clerck, 1757) \| 7 \|  \| 33 \|  \| \| Alopecosa trabalis (Clerck, 1757) \|  \| 1 \|  \|  \| \| Aphantaulax cincta (L. Koch, 1866) \|  \|  \| 2 \|  \| \| Araeoncus humilis (Blackwall, 1841) \|  \| 39 \|  \|  \| \| Araniella cucurbitina (Clerck, 1757) \|  \|  \| 1 \|  \| \| Argenna subnigra (O. P.-Cambridge, 1861) \|  \| 2 \|  \| 2 \| \| Argiope bruennichi (Scopoli, 1772) \|  \|  \| 1 \| 1 \| \| Asagena phalerata (Panzer, 1801) \| 2 \|  \| 3 \|  \| \| Aulonia albimana (Walckenaer, 1805) \|  \| 4 \| 37 \| 290 \| \| Bassaniodes robustus (Hahn, 1832) \|  \|  \| 2 \|  \| \| Bathyphantes gracilis (Blackwall, 1841) \| 1 \| 2 \|  \|  \| \| Ceratinella brevis (Wider, 1834) \|  \| 1 \|  \|  \| \| Ceratinella scabrosa (O. P.-Cambridge, 1871) \|  \| 1 \|  \|  \| \| Cercidia prominens (Westring, 1851) \|  \|  \|  \| 7 \| \| Civizelotes gracilis (Canestrini, 1868) \|  \| 2 \|  \|  \| \| Clubiona neglecta O. P.-Cambridge, 1862 \|  \| 1 \| 4 \| 3 \| \| Clubiona subtilis L. Koch, 1867 \|  \|  \|  \| 1 \| \| Clubiona terrestris Westring, 1851 \|  \|  \| 2 \|  \| \| Diaea dorsata (Fabricius, 1777) \| 2 \|  \| 2 \|  \| \| Dictyna arundinacea (Linnaeus, 1758) \| 1 \|  \| 6 \|  \| \| Dictyna pusilla Thorell, 1856 \|  \|  \| 1 \|  \| \| Dicymbium nigrum (Blackwall, 1834) \| 1 \| 1 \|  \|  \| \| Dicymbium tibiale (Blackwall, 1836) \| 1 \|  \| 1 \|  \| \| Diplocephalus cristatus (Blackwall, 1833) \|  \| 1 \|  \|  \| \| Diplostyla concolor (Wider, 1834) \| 11 \| 7 \| 4 \| 2 \| \| Dipoena coracina (C. L. Koch, 1837) \|  \|  \| 1 \|  \| \| Drassodes cupreus (Blackwall, 1834) \|  \|  \| 1 \|  \| \| Drassodes lapidosus (Walckenaer, 1802) \|  \|  \| 2 \|  \| \| Drassodes pubescens (Thorell, 1856) \| 1 \|  \| 2 \|  \| \| Drassyllus praeficus (L. Koch, 1866) \|  \| 6 \| 3 \| 2 \| \| Drassyllus pusillus (C. L. Koch, 1833) \| 1 \| 10 \|  \| 5 \| \| Drassyllus villicus (Thorell, 1875) \|  \| 1 \| 1 \|  \| \| Dysdera erythrina (Walckenaer, 1802) \|  \|  \| 5 \|  \| \| Enoplognatha latimana Hippa & Oksala, 1982 \|  \| 3 \|  \|  \| \| Enoplognatha thoracica (Hahn, 1833) \| 4 \| 3 \| 8 \|  \| \| Entelecara flavipes (Blackwall, 1834) \|  \|  \| 1 \|  \| \| Episinus truncatus Latreille, 1809 \|  \|  \| 14 \| 21 \| \| Erigone dentipalpis (Wider, 1834) \| 8 \| 446 \| 1 \|  \| \| Ero furcata (Villers, 1789) \|  \|  \| 3 \|  \| \| Euophrys frontalis (Walckenaer, 1802) \|  \|  \| 1 \|  \| \| Evarcha arcuata (Clerck, 1757) \| 2 \|  \| 3 \| 3 \| \| Evarcha laetabunda (C. L. Koch, 1846) \|  \|  \|  \| 1 \| \| Hahnia nava (Blackwall, 1841) \|  \| 1 \| 1 \|  \| \| Haplodrassus dalmatensis (L. Koch, 1866) \| 1 \| 1 \| 1 \|  \| \| Haplodrassus minor (O. P.-Cambridge, 1879) \| 3 \| 3 \| 3 \| 2 \| \| Haplodrassus signifer (C. L. Koch, 1839) \|  \| 1 \|  \|  \| \| Haplodrassus silvestris (Blackwall, 1833) \|  \|  \| 3 \|  \| \| Harpactea rubicunda (C. L. Koch, 1838) \|  \|  \| 7 \|  \| \| Heliophanus flavipes (Hahn, 1832) \|  \|  \| 2 \| 2 \| \| Histopona torpida (C. L. Koch, 1834) \|  \|  \| 1 \|  \| \| Hypsosinga pygmaea (Sundevall, 1832) \| 7 \| 6 \|  \|  \| \| Mangora acalypha (Walckenaer, 1802) \| 24 \| 13 \| 2 \| 4 \| \| Marpissa nivoyi (Lucas, 1846) \|  \|  \| 25 \| 10 \| \| Maso sundevalli (Westring, 1851) \|  \|  \| 4 \|  \| \| Mermessus trilobatus (Emerton, 1882) \| 1 \|  \|  \|  \| \| Micrargus subaequalis (Westring, 1851) \|  \| 1 \| 5 \|  \| \| Microdipoena jobi (Kraus, 1967) \|  \|  \| 1 \|  \| \| Microlinyphia impigra (O. P.-Cambridge, 1871) \|  \| 1 \|  \|  \| \| Microlinyphia pusilla (Sundevall, 1830) \|  \| 9 \|  \|  \| \| Minicia marginella (Wider, 1834) \|  \|  \| 4 \| 1 \| \| Myrmarachne formicaria (De Geer, 1778) \|  \| 1 \| 2 \| 6 \| \| Neottiura bimaculata (Linnaeus, 1767) \|  \| 10 \| 1 \| 19 \| \| Neottiura suaveolens (Simon, 1879) \| 7 \| 1 \| 5 \| 1 \| \| Neriene clathrata (Sundevall, 1830) \| 4 \|  \| 15 \|  \| \| Neriene furtiva (O. P.-Cambridge, 1870) \| 1 \| 1 \|  \| 1 \| \| Neriene montana (Clerck, 1757) \|  \| 1 \|  \| 1 \| \| Oedothorax apicatus (Blackwall, 1850) \| 1 \| 260 \|  \|  \| \| Oxyopes heterophthalmus Latreille, 1804 \| 2 \|  \| 1 \|  \| \| Ozyptila atomaria (Panzer, 1801) \|  \|  \|  \| 1 \| \| Ozyptila claveata (Walckenaer, 1837) \| 1 \|  \| 1 \|  \| \| Ozyptila praticola (C. L. Koch, 1837) \|  \|  \|  \| 1 \| \| Pachygnatha degeeri Sundevall, 1830 \| 149 \| 85 \| 151 \|  \| \| Palliduphantes insignis (O. P.-Cambridge, 1913) \|  \|  \| 1 \|  \| \| Pardosa agrestis (Westring, 1861) \| 1560 \| 2324 \| 381 \|  \| \| Pardosa hortensis (Thorell, 1872) \| 1 \| 12 \| 11 \| 1 \| \| Pardosa lugubris s.lat. (Walckenaer, 1802) \|  \| 1 \|  \|  \| \| Pardosa lugubris s.str. (Walckenaer, 1802) \| 7 \|  \| 209 \|  \| \| Pardosa paludicola (Clerck, 1757) \|  \| 6 \|  \|  \| \| Pardosa palustris (Linnaeus, 1758) \| 1 \| 4 \| 1 \| 1 \| \| Pardosa prativaga (L. Koch, 1870) \| 1 \| 11 \| 8 \| 2 \| \| Pardosa pullata (Clerck, 1757) \| 2 \| 3 \|  \| 1 \| \| Pardosa riparia (C. L. Koch, 1833) \|  \|  \| 2 \|  \| \| Philodromus cespitum (Walckenaer, 1802) \|  \|  \|  \| 1 \| \| Phlegra fasciata (Hahn, 1826) \|  \| 1 \|  \|  \| \| Phrurolithus festivus (C. L. Koch, 1835) \| 1 \| 3 \| 16 \| 9 \| \| Phylloneta impressa (L. Koch, 1881) \|  \| 1 \|  \|  \| \| Pisaura mirabilis (Clerck, 1757) \| 70 \| 40 \| 39 \| 27 \| \| Pocadicnemis juncea Locket & Millidge, 1953 \|  \| 1 \|  \|  \| \| Porrhomma microphthalmum (O. P.-Cambridge, 1871) \|  \| 7 \|  \|  \| \| Pseudeuophrys obsoleta (Simon, 1868) \|  \|  \|  \| 1 \| \| Robertus arundineti (O. P.-Cambridge, 1871) \|  \| 5 \| 4 \|  \| \| Robertus lividus (Blackwall, 1836) \|  \| 1 \|  \|  \| \| Sibianor aurocinctus (Ohlert, 1865) \|  \|  \|  \| 1 \| \| Silometopus elegans (O. P.-Cambridge, 1872) \| 1 \|  \| 1 \|  \| \| Singa nitidula C. L. Koch, 1844 \|  \| 1 \|  \|  \| \| Synema globosum (Fabricius, 1775) \|  \| 1 \|  \|  \| \| Talavera aequipes (O. P.-Cambridge, 1871) \| 1 \|  \|  \|  \| \| Talavera aperta (Miller, 1971) \|  \|  \|  \| 1 \| \| Talavera monticola (Kulczynski, 1884) \|  \|  \|  \| 1 \| \| Tegenaria agrestis (Walckenaer, 1802) \| 1 \|  \| 11 \|  \| \| Tenuiphantes tenuis (Blackwall, 1852) \| 3 \| 17 \|  \| 1 \| \| Thanatus formicinus (Clerck, 1757) \|  \|  \| 2 \|  \| \| Thanatus striatus C. L. Koch, 1845 \|  \| 1 \|  \|  \| \| Tibellus oblongus (Walckenaer, 1802) \| 3 \| 78 \| 2 \| 264 \| \| Tmarus piger (Walckenaer, 1802) \| 1 \|  \|  \|  \| \| Trichoncus affinis Kulczynski, 1894 \|  \|  \| 1 \|  \| \| Trochosa hispanica Simon, 1870 \| 1 \|  \|  \|  \| \| Trochosa robusta (Simon, 1876) \| 2 \|  \| 15 \|  \| \| Trochosa ruricola (De Geer, 1778) \| 28 \| 5 \| 14 \|  \| \| Trochosa terricola Thorell, 1856 \| 8 \| 3 \| 6 \| 1 \| \| Urocoras longispina (Kulczynski, 1897) \|  \|  \|  \| 1 \| \| Xerolycosa miniata (C. L. Koch, 1834) \| 4 \| 3 \| 16 \|  \| \| Xysticus bifasciatus C. L. Koch, 1837 \| 2 \|  \| 6 \| 1 \| \| Xysticus cristatus (Clerck, 1757) \|  \|  \| 3 \| 1 \| \| Xysticus kochi Thorell, 1872 \| 10 \| 11 \| 10 \|  \| \| Zelotes electus (C. L. Koch, 1839) \|  \|  \| 1 \|  \| \| Zelotes longipes (L. Koch, 1866) \| 1 \|  \|  \|  \| \| Zelotes oblongus (C. L. Koch, 1833) \|  \|  \| 1 \|  \| \| Zora armillata Simon, 1878 \|  \|  \|  \| 2 \| \| Zora parallela Simon, 1878 \|  \| 1 \|  \|  \| \| Zora spinimana (Sundevall, 1833) \| 2 \|  \| 30 \| 1 \| |
| --- | --- | --- | --- | --- | --- | --- | --- | --- | --- | --- | --- | --- | --- | --- | --- | --- | --- | --- | --- | --- | --- | --- | --- | --- | --- | --- | --- | --- | --- | --- | --- | --- | --- | --- | --- | --- | --- | --- | --- | --- | --- | --- | --- | --- | --- | --- | --- | --- | --- | --- | --- | --- | --- | --- | --- | --- | --- | --- | --- | --- | --- | --- | --- | --- | --- | --- | --- | --- | --- | --- | --- | --- | --- | --- | --- | --- | --- | --- | --- | --- | --- | --- | --- | --- | --- | --- | --- | --- | --- | --- | --- | --- | --- | --- | --- | --- | --- | --- | --- | --- | --- | --- | --- | --- | --- | --- | --- | --- | --- | --- | --- | --- | --- | --- | --- | --- | --- | --- | --- | --- | --- | --- | --- | --- | --- | --- | --- | --- | --- | --- | --- | --- | --- | --- | --- | --- | --- | --- | --- | --- | --- | --- | --- | --- | --- | --- | --- | --- | --- | --- | --- | --- | --- | --- | --- | --- | --- | --- | --- | --- | --- | --- | --- | --- | --- | --- | --- | --- | --- | --- | --- | --- | --- | --- | --- | --- | --- | --- | --- | --- | --- | --- | --- | --- | --- | --- | --- | --- | --- | --- | --- | --- | --- | --- | --- | --- | --- | --- | --- | --- | --- | --- | --- | --- | --- | --- | --- | --- | --- | --- | --- | --- | --- | --- | --- | --- | --- | --- | --- | --- | --- | --- | --- | --- | --- | --- | --- | --- | --- | --- | --- | --- | --- | --- | --- | --- | --- | --- | --- | --- | --- | --- | --- | --- | --- | --- | --- | --- | --- | --- | --- | --- | --- | --- | --- | --- | --- | --- | --- | --- | --- | --- | --- | --- | --- | --- | --- | --- | --- | --- | --- | --- | --- | --- | --- | --- | --- | --- | --- | --- | --- | --- | --- | --- | --- | --- | --- | --- | --- | --- | --- | --- | --- | --- | --- | --- | --- | --- | --- | --- | --- | --- | --- | --- | --- | --- | --- | --- | --- | --- | --- | --- | --- | --- | --- | --- | --- | --- | --- | --- | --- | --- | --- | --- | --- | --- | --- | --- | --- | --- | --- | --- | --- | --- | --- | --- | --- | --- | --- | --- | --- | --- | --- | --- | --- | --- | --- | --- | --- | --- | --- | --- | --- | --- | --- | --- | --- | --- | --- | --- | --- | --- | --- | --- | --- | --- | --- | --- | --- | --- | --- | --- | --- | --- | --- | --- | --- | --- | --- | --- | --- | --- | --- | --- | --- | --- | --- | --- | --- | --- | --- | --- | --- | --- | --- | --- | --- | --- | --- | --- | --- | --- | --- | --- | --- | --- | --- | --- | --- | --- | --- | --- | --- | --- | --- | --- | --- | --- | --- | --- | --- | --- | --- | --- | --- | --- | --- | --- | --- | --- | --- | --- | --- | --- | --- | --- | --- | --- | --- | --- | --- | --- | --- | --- | --- | --- | --- | --- | --- | --- | --- | --- | --- | --- | --- | --- | --- | --- | --- | --- | --- | --- | --- | --- | --- | --- | --- | --- | --- | --- | --- | --- | --- | --- | --- | --- | --- | --- | --- | --- | --- | --- | --- | --- | --- | --- | --- | --- | --- | --- | --- | --- | --- | --- | --- | --- | --- | --- | --- | --- | --- | --- | --- | --- | --- | --- | --- | --- | --- | --- | --- | --- | --- | --- | --- | --- | --- | --- | --- | --- | --- | --- | --- | --- | --- | --- | --- | --- | --- | --- | --- | --- | --- | --- | --- | --- | --- | --- | --- | --- | --- | --- | --- | --- | --- | --- | --- | --- | --- | --- | --- | --- | --- | --- | --- | --- | --- | --- | --- | --- | --- | --- | --- | --- | --- | --- | --- | --- | --- | --- | --- | --- | --- | --- | --- | --- | --- | --- | --- | --- | --- | --- | --- | --- | --- | --- | --- | --- | --- | --- | --- | --- | --- | --- | --- | --- | --- | --- | --- | --- | --- | --- | --- | --- | --- | --- | --- | --- | --- | --- | --- | --- | --- | --- | --- | --- | --- | --- | --- | --- | --- | --- | --- | --- | --- | --- | --- | --- | --- | --- | --- | --- | --- | --- | --- | --- | --- | --- | --- | --- | --- | --- | --- | --- | --- |
